# Draft Whole-Genome Sequence of a New Variant of *Vibrio cholerae* O1 El Tor Strain Isolated from a Cholera Patient in Russia

**DOI:** 10.1128/genomeA.00432-14

**Published:** 2014-05-29

**Authors:** Nina I. Smirnova, Alexander V. Cherkasov, Yaroslav M. Krasnov, Dmitriy A. Agafonov, Vladimir V. Kutyrev

**Affiliations:** Federal Government Health Institution Russian Researсh Anti-Plaque Institute Microbe, Saratov, Russia

## Abstract

Draft whole-genome sequencing of the *Vibrio cholerae* О1 El Tor clinical strain L3226, isolated in Moscow in 2010, was carried out. Various mutations in the virulence-associated mobile elements were determined in its genome that differentiated this strain from the reference *V. cholerae* О1 El Tor strain N16961*.*

## GENOME ANNOUNCEMENT

*Vibrio cholerae* is the causative agent of cholera, a severe diarrheal disease that continues to be a major public health problem in many developing countries. Epidemic outbreaks of cholera have been caused by only two *V. cholerae* biotypes: classical and El Tor (1). Since the early 1990s, new variants of *V. cholerae* О1 El Tor that possess traits of both the classical and El Tor biotypes have emerged (2, 3). These strains cause more severe cholera, with higher fatality rates, than that caused by prototypic El Tor strains.

Here, we report the first draft whole-genome sequence of a clinical strain, *V. cholerae* O1 El Tor L3226, isolated in 2010 in Moscow, and we compared it to the complete genome of the reference strain *V. cholerae* O1 El Tor N16961.

Libraries for sequencing were prepared from 1 µg of purified genomic DNA, according to the manufacturer’s protocol, and were sequenced on a PGM platform (Life Technologies, CA) using 318 Chip and 200-bp chemistry. Raw single-end reads (2,287,084 total) were trimmed using the Ion Torrent Suite Software and were assembled *de novo* using the Newbler GS Assembler version 2.6 (Roche). Gene prediction, annotation, and an RNA search were performed by Rapid Annotations using Subsystems Technology (RAST) (4).

The draft genome sequence of *V. cholerae* L3226 was assembled into 121 contigs (86-fold coverage) with a total length of 3,991,045 bp and an average G+C content of 47.4%. The *N*_50_ of the contigs is 134 kb, with the longest contig being 329 kb. The annotation of the obtained contigs showed the presence of 3,917 open reading frames (ORFs), 3,852 protein-coding sequences, and 65 tRNAs. The *V. cholerae* L3226 contigs were mapped into two distinct chromosomes, as in typical *V. cholerae* strains. Chromosome I consists of 47 contigs, amounting to a total length of 2,978,616 bp. Chromosome II consists of 44 contigs, with a total length of 1,007,260 bp. The chromosome size and G+C content correlate well with those of other *V. cholerae* strains (5).

The sequenced genome analysis revealed that the nucleotide sequences of virulence-associated mobile elements, such as CTXφ prophage, carrying the cholera toxin (CT)-coding genes *ctxAB*, *Vibrio* pathogenicity island 1 (VPI-1), and *Vibrio* seventh pandemic island II (VSP-II) differed from those of the reference strain N16961. Simultaneously, these regions of strain L3226 have high similarity to the same genome regions of the new El Tor variants isolated in India, Bangladesh, and Haiti (6–8). Interestingly, in contrast to El Tor variants possessing *ctxAB* (*ctxB1* allele) of the reference classical strains, the *ctxB1* gene of L3226 strain carried a novel mutation, a C→A substitution in position 20. Another peculiarity of the L3226 strain is found in a 13-kb deletion in the VSP-II. In view of the recent discovery of new variants of *V. cholerae* El Tor, the sequenced genome of L3226 strain might provide further opportunity to investigate the evolution of cholera agents in the modern period.

### Nucleotide sequence accession number.

This whole-genome shotgun project for the L3226 strain has been deposited at DDBJ/EMBL/GenBank under the accession no. JDVX01000000. The version presented in this article is the first one.
